# New insights into hepatitis B virus lymphotropism: Implications for HBV-related lymphomagenesis

**DOI:** 10.3389/fonc.2023.1143258

**Published:** 2023-03-15

**Authors:** Valentina Svicher, Romina Salpini, Stefano D’Anna, Lorenzo Piermatteo, Marco Iannetta, Vincenzo Malagnino, Loredana Sarmati

**Affiliations:** ^1^Department of Experimental Medicine, University of Rome Tor Vergata, Rome, Italy; ^2^Department of Biology, University of Rome Tor Vergata, Rome, Italy; ^3^Clinical Infectious Diseases, Department of System Medicine, University of Rome Tor Vergata, Rome, Italy

**Keywords:** hepatitis B virus, HBV, carcinogenesis, lymphotropism, lymphomagenesis, tumors

## Abstract

HBV is one of the most widespread hepatitis viruses worldwide, and a correlation between chronic infection and liver cancer has been clearly reported. The carcinogenic capacity of HBV has been reported for other solid tumors, but the largest number of studies focus on its possible lymphomagenic role. To update the correlation between HBV infection and the occurrence of lymphatic or hematologic malignancies, the most recent evidence from epidemiological and *in vitro* studies has been reported. In the context of hematological malignancies, the strongest epidemiological correlations are with the emergence of lymphomas, in particular non-Hodgkin’s lymphoma (NHL) (HR 2.10 [95% CI 1.34-3.31], p=0.001) and, more specifically, all NHL B subtypes (HR 2.14 [95% CI 1.61-2.07], p<0.001). Questionable and unconfirmed associations are reported between HBV and NHL T subtypes (HR 1.11 [95% CI 0.88-1.40], p=0.40) and leukemia. The presence of HBV DNA in peripheral blood mononuclear cells has been reported by numerous studies, and its integration in the exonic regions of some genes is considered a possible source of carcinogenesis. Some *in vitro* studies have shown the ability of HBV to infect, albeit not productively, both lymphomonocytes and bone marrow stem cells, whose differentiation is halted by the virus. As demonstrated in animal models, HBV infection of blood cells and the persistence of HBV DNA in peripheral lymphomonocytes and bone marrow stem cells suggests that these cellular compartments may act as HBV reservoirs, allowing replication to resume later in the immunocompromised patients (such as liver transplant recipients) or in subjects discontinuing effective antiviral therapy. The pathogenetic mechanisms at the basis of HBV carcinogenic potential are not known, and more in-depth studies are needed, considering that a clear correlation between chronic HBV infection and hematological malignancies could benefit both antiviral drugs and vaccines.

## Introduction

Several viruses, often causing chronic or persistent infections, are correlated with the occurrence of malignancy. Usually, the immune response, with its articulated actions, represents the best control against the emergence of neoplastic cells; however, the persistence of viruses with oncogenic characteristics represents the driver for immune evasion, facilitating poor control over malignancy emergence.

Epstein−Barr virus, human papillomavirus, human herpesvirus-8, Merkel cell polyomavirus, and hepatitis B viruses (HBV) and hepatitis C virus (HCV) are commonly considered viruses with oncogenic properties. The correlation between these infections and the emergence of cancer has been clearly demonstrated; although, the development of malignancy is not common among infected subjects (1, 2). Indeed, multiple additional factors, such as chronic inflammation, environmental mutagens, or immunosuppression, are required for cancer development; therefore, some virus-related tumors are more common in immunocompromised patients (solid organ or bone marrow transplant recipients, human immunodeficiency virus [HIV]-infected patients) and in patients with other chronic inflammatory conditions.

HBV is one of the most widespread hepatitis viruses worldwide, with 296 million infected subjects, including over 6 million children under the age of 5 (3). The correlation between chronic HBV infection and liver cancer is widely known, and it has been reported that more than 80% of hepatocellular carcinoma (HCC) diagnoses are related to HBV infection (4). However, HCC is not the only malignancy correlated with HBV infection, as a few other non-hepatic malignancies have been associated with the presence of chronic HBV infection. A recent study on 496,732 Chinese participants (5) assessed the associations between chronic HBV infection and the risk of all cancer types, and the authors found an increased risk of developing several non-liver cancers, including stomach, oral, colorectal, and pancreatic cancers, and lymphomas.

The positive correlation between chronic HBV infection and the emergence of lymphomas has been repeatedly reported in the literature. However, although several meta-analyses reported a positive correlation (6), others did not (7); therefore, the topic remains still dibated.

The purpose of this review is to provide information on HBV lymphotropism and its potential impact on lymphomagenesis. With this aim, the epidemiological correlates between HBV infection and the occurrence of lymphatic or hematological tumors have been updated, and the results of *in vitro* studies of HBV infection of hematopoietic and lymphatic cells have been reported, suggesting possible patterns of persistent infection and viral reactivation. Finally, some considerations on possible implications between HBV infection of blood cells and the possibility of new targets for eradication treatments are reported.

## Correlation between HBV infection and hematological disorders: Data from epidemiological studies

The role of HBV in the field of oncogenesis is mainly related to the occurrence of HCC, and some studies have evaluated the protective effect of HBV vaccination and antiviral drugs on HCC emergence (8, 9). The association between HBV and HCC is directly related to HBV’s hepatotropic characteristics and its ability to infect and replicate inside hepatocyte cells. However, it is now known (10–12) that HBV can also infect other cells from extrahepatic sites, such as lymph nodes, spleen, thyroid, kidneys, and pancreas, and its influence on extrahepatic tumor emergence has been studied. HBV replicates in hematopoietic and lymphoid cells and their progenitors (10, 13), making this compartment an additional reservoir of infection and suggesting an implication of the virus in lymphomagenesis (**Table 1**).

**Table 1:** Summary of studies addressing the correlation between HBV infection and hematological disorders.

Authors	Country	High HBV prevalence	Study characteristic	Results	Reference
Song C. et al	China	Yes	Prospective study cohort n. of patients: 496732	Lymphoma in HBsAg-positive patients: IR 0.08 x 1000 P/Y, aHR 2.10 [95% CI 1.34-3.31], p=0.001. No HBV correlation with leukemia	(5)
Kamiza A.B. et al.	Taiwan	Yes	Prospective study cohort n.of patients HBV 15888	HR LNH in HBsAg-positive patients 2.10, 95% CI 1.25-3.52	(14)
Zhou X. et al.	China	Yes	Case-control study 3502 NHL patients (23 [14.9%] patients HBsAg-positive) 7004 control patients	B-NHL in HBsAg-positive patients: AOR 2.14 (CI 95% 1. 88–2.45) DLBCL in HBsAg-positive patients: AOR 2.45 (CI: 2.07–2.89) T-NHL in HBsAg positive patients AOR not significant: HR 1.11 (CI 0.88-1.40), p=0.40	(15)
Engels E.A. et al.	South Korea	Yes	Cohort Study 53,045 subjects, 8.8% HBsAg positive	NHL in HBsAg positive patients overall incidence 19.4 vs. 12.3 per 100,000 person-years; aHR 1.74, CI:1.45–2.09 DLBCL in HBsAg positive patients aHR 2.01, CI 1.48– 2.75	(16)
Yood M.U. et al.	U.S.A.	No	Cohort Study 3888HBsAg positive patients	NHL in HBsAg positive patients: aHR 2.80, 95% 1.16-6.75	(17)
Andersen E.S. et al.	Denmark	No	Cohort Study 4345 HBsAg positive patients	NHL in HBsAg positive patients IC 1.22 (CI 0.42–3.55)	(18)

IC, incidence rate; aHR, adjusted Hazard Ratio; AOR, Adjusted Odds Ratio; B-NHL, B non Hodgkin Lymphoma; T-NHL, T Non Hodgkin Lymphoma; DLBCL, Diffuse large B-cell lymphoma.

Some recent studies have attempted to evaluate the impact of active HBV infection on the onset of all types of extrahepatic cancer (5). In a prospective cohort study by Ci Song et al. found a significant correlation for the occurrence of lymphoma, but not leukemia in HBV patients. Recent studies have confirmed the absence of a role for HBV in the pathogenesis of leukemia, such as the case–control study by Kamiza et al. in Taiwan (14) and the South Korean cohort study by Engels (16), which also distinguished different subtypes of leukemia (including multiple myeloma [MM]; lymphoid leukemia; myeloid/monocytic leukemia).

Lymphomas are the hematological tumors most epidemiologically associated with HBV infection (5, 14). Not all varieties of lymphoma were associated with active HBV infection, and Kamiza et al. (14) showed a two fold increase of risk for non-Hodgkin’s lymphoma (NHL) in the HBV-positive population.

A recent Chinese paper by Zhou et al. (15) investigated the impact of HBV on the onset of NHL, specifying the different subtypes of the onco-hematological pathology, given that the frequency of the major NHL subtypes varies substantially by geographic region (19). In this study, B-cell NHL (B-NHL) and T-cell NHL (T-NHL) were distinguished, and all further subtypes were considered. Diffuse large B-cell lymphoma (DLBCL) was the most common (n=1224 patients) and thus was further distinguished into germinal center-type lymphoma (DLBCL-GCB n=356) or non-GCB-DLBCL (n=663). Interestingly, 17% of patients with B-NHL were HBsAg-positive, while 9.4% of T-NHL patients were positive. Indeed, the association between HBV infection and the occurrence of B-NHL was statistically significant in multivariate analysis (adjusted for age, sex, and year of diagnosis) and a doubled risk of occurrence of B-NHL has been described yet. However, the association between HBV and the onset of T-NHL was not significant. The majority of B-NHL subtypes were found to be associated with HBV infection: a more than doubled risk has been identified in DLBCL, follicular lymphoma (FL), Small lymphocytic lymphoma/chronic lymphocytic leukemia (SLL/CLL) and precursor B lumphoblastic lymphoma. No differences were found between GCB-type and non-GCB-type patients. Furthermore, only two subtypes of T-NHL were found to be significantly associated with HBV infection: angioimmunoblastic T-cell lymphoma (AITL) and anaplastic large cell lymphoma (ALCL). Among other cohort studies (8, 16–18), only the Danish DANVIR study did not find statistical significance in the association between NHL and HBV infection and, consequently, with the different subtypes of NHL. This can be related to HBV epidemiological data of Denmark, which is a country with low HBV prevalence, with main sexual transmission and limited chronic HBV infection.

The influence of anti-HBV antiviral treatments and vaccinations on lymphomagenesis was also studied. Both variables are absent in most of the above cited study cohorts. One of the most relevant studies on this issue is that of Huang et al. (20) on a Taiwanese study cohort, which covered the 17-year period from 1997 to 2013 and compared the incidence of NHL in HBV-positive and HBV-negative groups. History of anti-HBV treatment, including interferon, adefovir, entecavir, lamivudine and tenofovir, was recorded, as well as the diagnosis of NHL based on The National Health Insurance Research Database (NHIRD). The study showed that only 3 of 4,069 (0.07%) patients developed NHL in the follow-up period, significantly less than the 107 of 34,559 (0.31%) patients without anti-HBV treatment (p = 0.0076) in the HBV cohort, but it was only a prevalence analysis with a lack of clinical and virological data at the time of diagnosis and during follow-up. In the same study, a significantly lower incidence of NHL was found in the group born after July 1984 (introduction of universal vaccination in Taiwan) as compared to the group born before 1984 (0.74 [95% CI 0.41-1.34] vs. 1.85 [95% CI 1.02-3.24], respectively, p=0.032) between the ages of 12.5 and 20 years but was not confirmed for the group aged 21 to 29.5 years. Therefore, the authors concluded that the definite impact of HBV vaccination in the population older than 21 years needs a much longer follow-up and should be adjusted for increasing incidence of NHL in the general population, even under the assumption that vaccination has a protective effect on developing NHL in the population younger than 20 years.

In conclusion, most cohort studies conducted in high HBV-prevalence countries have demonstrated a correlation between HBV infection and NHL, while the impact of HBV on the onset of leukemia, Hodgkin’s lymphoma and other hematological disorders is controversial. New studies are needed, including prospective clinical and virological studies that involve control groups, which are often lacking in retrospective and prospective epidemiological studies. Moreover, longer follow-up studies would allow us to better evaluate the influence of vaccination status and antiviral therapy on cancer development.

## HBV DNA integration in immune cells as a mechanism mediating lymphomagenesis

The integration of HBV DNA into the genome of human hepatocytes is a recognized mechanism contributing to the onset of HCC (21). HBV DNA integration can promote the neoplastic transformation of the involved hepatocytes by several mechanisms, such as the direct dysregulation of oncogenes and onco-suppressors, the production of chimeric viral-human RNAs and proteins with transactivating properties, and the promotion of overall genome instability, further enhancing the accumulation of chromosomal aberrations (22–28). Overall, these mechanisms can trigger the clonal selection of hepatocytes with enhanced survival and proliferation, promoting their neoplastic transformation (23, 25, 29–31).

**Figure 1:**
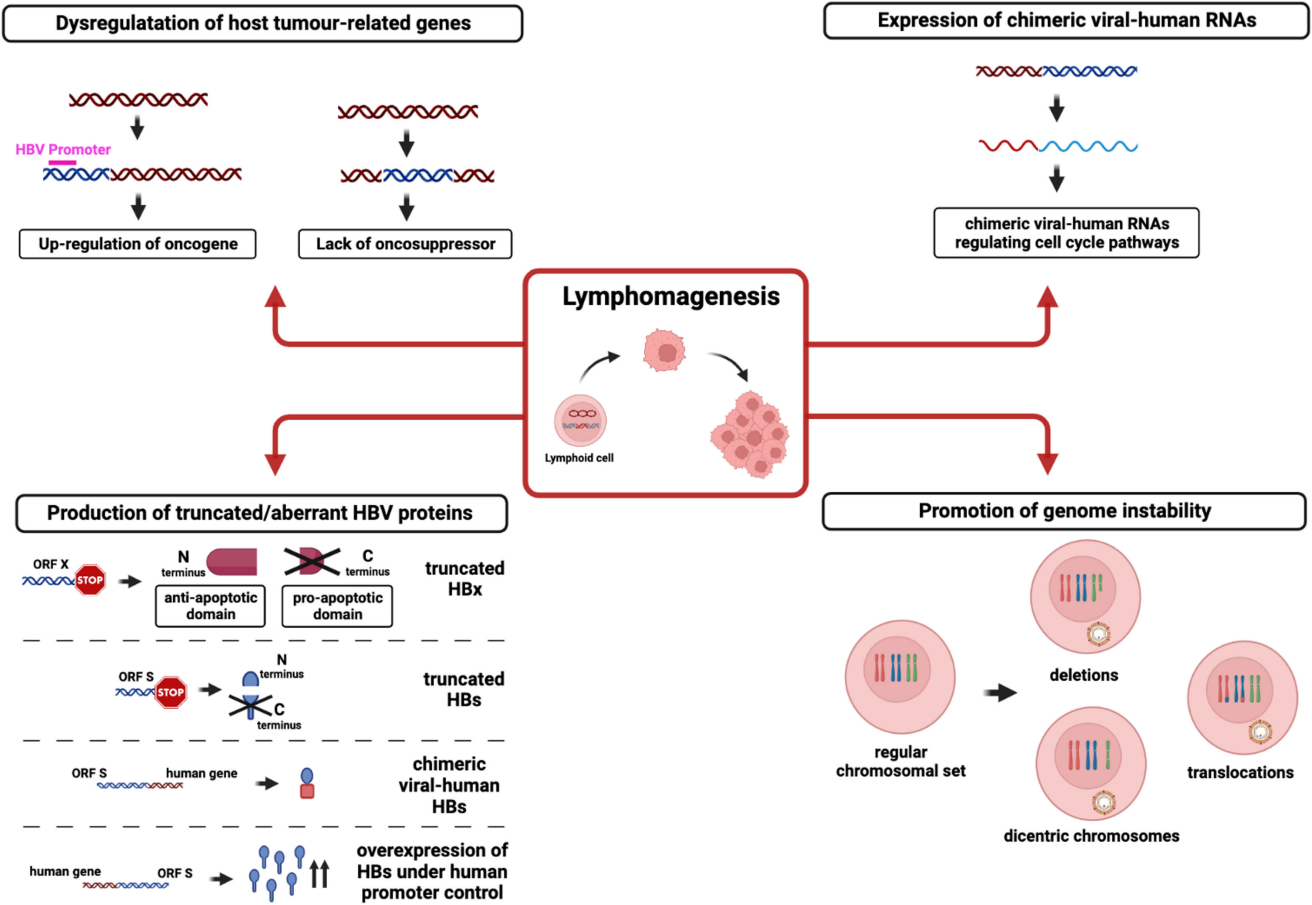
Mechanisms involved in lymphomagenesis caused by HBV DNA integration into the host genome. The figure shows the potential pathways by which HBV integration in lymphoid cell genome leads to lymphomagenesis: i) HBV DNA (blue) integration into the infected cells’ genome (red) can alter host tumor-related genes by up-regulating oncogene or by interrupting oncosuppressor activity; ii) HBV integration can generate chimeric viral-human RNAs with a role in regulating cell cycle pathways; iii) integrated HBV DNA can lead to the production of truncated/aberrant HBV proteins, (such as truncated HBx or HBs) as well as chimeric viral-human proteins; iv) HBV integration can promote genomic instability by altering the regular chromosomal set.

In a similar fashion, some recent studies have also supported the role of HBV DNA integration into the genome of immune cells in driving the pathogenesis of immunoproliferative disorders such as NHL and leukemia (32) (**Figure 1**). This hypothesis is in keeping with previous studies demonstrating the occurrence of HBV DNA integration into the genome of peripheral blood mononuclear cells (PBMCs) from patients with chronic HBV infection (33–36) and is also in line with data from the woodchuck model identifying multiple hepadnaviral integrations not only in PBMCs but also in lymphatic organs, including bone marrow, spleen, and lymph nodes (37).

In this regard, a recent study revealed that HBV DNA integration represents a common phenomenon in NHL tissues of patients with HBV infection (38). Indeed, multiple HBV DNA integration events have been observed in half of the HBsAg-positive patients with NHL analyzed in this study. Notably, HBV DNA integration was found in the exonic regions (crucial for mRNA synthesis) of four specific genes (FAT2, SsTX, ITGA10, and CD63), determining their altered expression and potentially perturbing relevant intracellular pathways. Similarly, HBV DNA integration was also recurrently found in seven coding genes (ANKS1B, CAPZB, CTNNA3, EGFLAM, FHOD3, HDAC4, and OPCML) potentially involved in mechanisms underlying carcinogenesis. Of note, six of these seven genes, preferentially targeted by HBV DNA integration, showed increased expression in NHL with respect to normal lymphatic tissues, suggesting that HBV DNA integration could promote lymphomagenesis mostly by cis-activating pro-oncogenes (38).

In line with these data, another study has recently shown that HBV DNA integration profiles in PBMCs are superimposable to those observed in tumor liver tissues, since they mostly involve genes regulating cell survival and proliferation in both body compartments. This study also provided evidence of HBV DNA integration within an extrahepatic lymphoproliferative tumor in a patient with chronic hepatitis B. Overall findings further reinforce the role of HBV DNA integration in PBMCs in paving the way toward lymphomagenesis (39).

Beyond PBMCs, HBV DNA integration has also been detected in the bone marrow hematological stem cells (HSCs) of chronically infected HBV patients (40). These integrations in the precursors of immune cells could promote uncontrolled cell proliferation and, in turn, could represent a mechanism favoring the onset of different types of lymphoproliferative disorders.

Overall, further studies are necessary to better explore the functional consequences of HBV DNA integration on immune cell function, malignant cell surveillance, and oncogenic transformation to finally elucidate the role of HBV DNA integration in immune cells and their precursors in hematological malignancies. Similarly, the limited knowledge of the kinetics, frequencies and specific mechanisms underlying HBV DNA integration in immune cells calls for further investigations to unveil these relevant issues.

## Evidence of HBV infection in hematopoietic stem cells and lymphoid cells in *in vitro* studies and animal models

**Figure 2:**
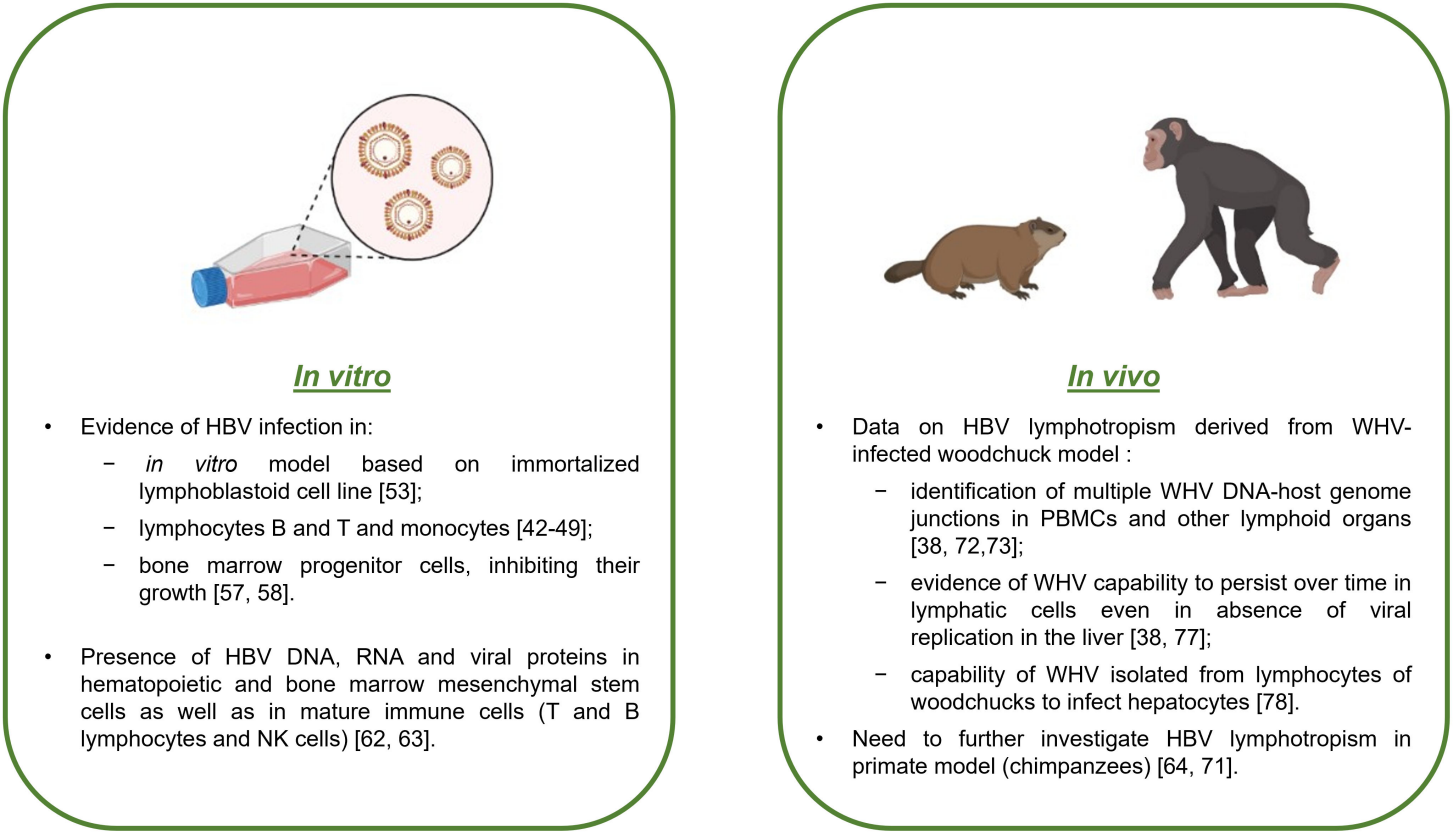
Evidence of HBV infection in lymphoid cells from *in vitro* and *in vivo* studies. Schematic summary with the main results of HBV infection in lymphoid cells based on cell cultures and animal models.

Early studies after the discovery of HBV included poor information on the capability of HBV to infect, persist and propagate in cells other than hepatocytes, mainly due to limitations of the techniques employed at that time (32). Few data are present in the literature on *in vitro* models of HBV infection, especially in blood mononuclear cells. However, over the years, several studies have demonstrated the presence of viral genomes or other replicative intermediates in cells such as B and T lymphocytes and monocytes (41–48) (**Figure 2**). Nevertheless, the mechanism of HBV entry into immune cells is still unknown, as it is unlikely that the sodium taurocholate cotransporting polypeptide (NTCP) receptor mediating viral entry into human hepatocytes is involved (32).

Although hepatocytes represent an eligible *in vitro* model for studying HBV infection (49–51), primary human hepatocytes are difficult to obtain, and consequently, it is difficult to study HBV infection in this kind of cell culture (51). However, since the end of the 1980s, it has been possible to create an *in vitro* model of an immortalized lymphoblastoid cell line sensitive to HBV infection, which is very useful for understanding the mechanisms of viral infection, replication, and propagation (52) (**Figure 2**). Early *in vitro* studies on HBV infection in PBMCs demonstrated that HBV was able to bind to the cell surface but was not able to completely infect or replicate inside PBMCs by evaluating the presence of cccDNA with the classical PCR method (51). From a methodological aspect, when analyzing HBV infection in *in vitro* models of PBMCs, it is important to be sure that the positivity of viral DNA effectively comes from the infected cells and not from viral particles or virus-free DNA fragments. Therefore, limited digestion of cell surfaces with trypsin and DNase and deep washing prior to nucleic acid extraction become crucial (33, 53, 54). The final confirmation of HBV lymphotropism is provided by the capability of immune cells to produce infectious virus (32).

One of the first findings resulting from *in vitro* studies on HBV infection in lymphatic cells was the capability of HBV to infect bone marrow progenitor cells and inhibit their growth (48, 55) (**Figure 2**). More specifically, only viral particles containing HBV DNA were infectious and capable of causing cell growth inhibition, unlike sub-viral particles or heat-inactivated virions, which had no effect on progenitor stem cells (48, 55).

Many studies have tested the susceptibility of healthy volunteer-derived hematopoietic and mesenchymal stem cells to HBV infection after exposure to HBV-positive sera (40–42, 48, 56–58). Positive results for intracellular HBV DNA and/or RNA and HBsAg were found in CD34+ hematopoietic stem cells (precursors of immune cells, myeloid and erythroid cell linages) (56, 57), as well as in bone marrow mesenchymal stem cells reactive to CD105 and CD90. Notably, the latter also represent hepatocyte progenitors (58).

Moreover, HBV lymphotropism was also demonstrated in mature immune cells (**Figure 2**). In particular, HBV mRNAs were detected in both B- and T-cell subsets with a higher degree in B lymphocytes (59). Another study revealed the presence of viral genome and proteins in NK cells (60) (**Figure 2**).

Regarding *in vivo* models, many studies have demonstrated the presence of HBV infection in extrahepatic sites, such as PBMCs, spleen, pancreas, and kidney, in animal models (46, 61–67). Notably, various animals can be used for studying HBV infection, but chimpanzees represent the most suitable model, as they can be infected by human HBV and present many useful characteristics, offering the opportunity to study different aspects of viral infection and its consequences (67) (**Figure 2**). Nonetheless, their use for research purposes is limited by handling due to the large size of the animals, ethical issues, and high costs (67). HBV DNA integration into the genome of PBMCs of chronically infected HBV chimpanzees was demonstrated (61).

Woodchucks represent another interesting model, where it was possible to detect woodchuck hepatitis virus (WHV) in immune cells (32) (**Figure 2**). In particular, the study’s results showed that immune cells support productive replication of WHV and that the derived viral progeny is infectious to hepatocytes and to immune cells in *in vitro* and *in vivo* models, where it can cause hepatitis (32). In the woodchuck model, multiple WHV DNA-host genome junctions were identified in PBMCs and other lymphoid organs, regardless of whether HCC had developed (37, 68, 69) (**Figure 2**). However, further studies are needed to understand the role of these WHV DNA integration events in PBMCs in lymphoproliferative disorders, such as NHL and chronic lymphocytic leukemia (34, 70, 71). Another important aspect, demonstrated in the woodchuck model, was the capability of WHV to persist in lymphatic cells without involving the liver (37, 72), representing a crucial point for understanding how this mechanism could be involved in viral persistence and likely consequent reactivation (**Figure 2**). Furthermore, it was demonstrated that in infected woodchucks, lymphotropic WHV was also able to infect hepatocytes and that the viral genomic sequences revealed in both types of cells were the same (73), underlining how lymphotropism could be a natural property of wild-type WHV and not a consequence of the development of lymphotropic variants (**Figure 2**).

## HBV infection in lymphoid cells as a novel route of HBV persistence, a source of HBV reactivation and a site for HBV genetic evolution

**Figure 3:**
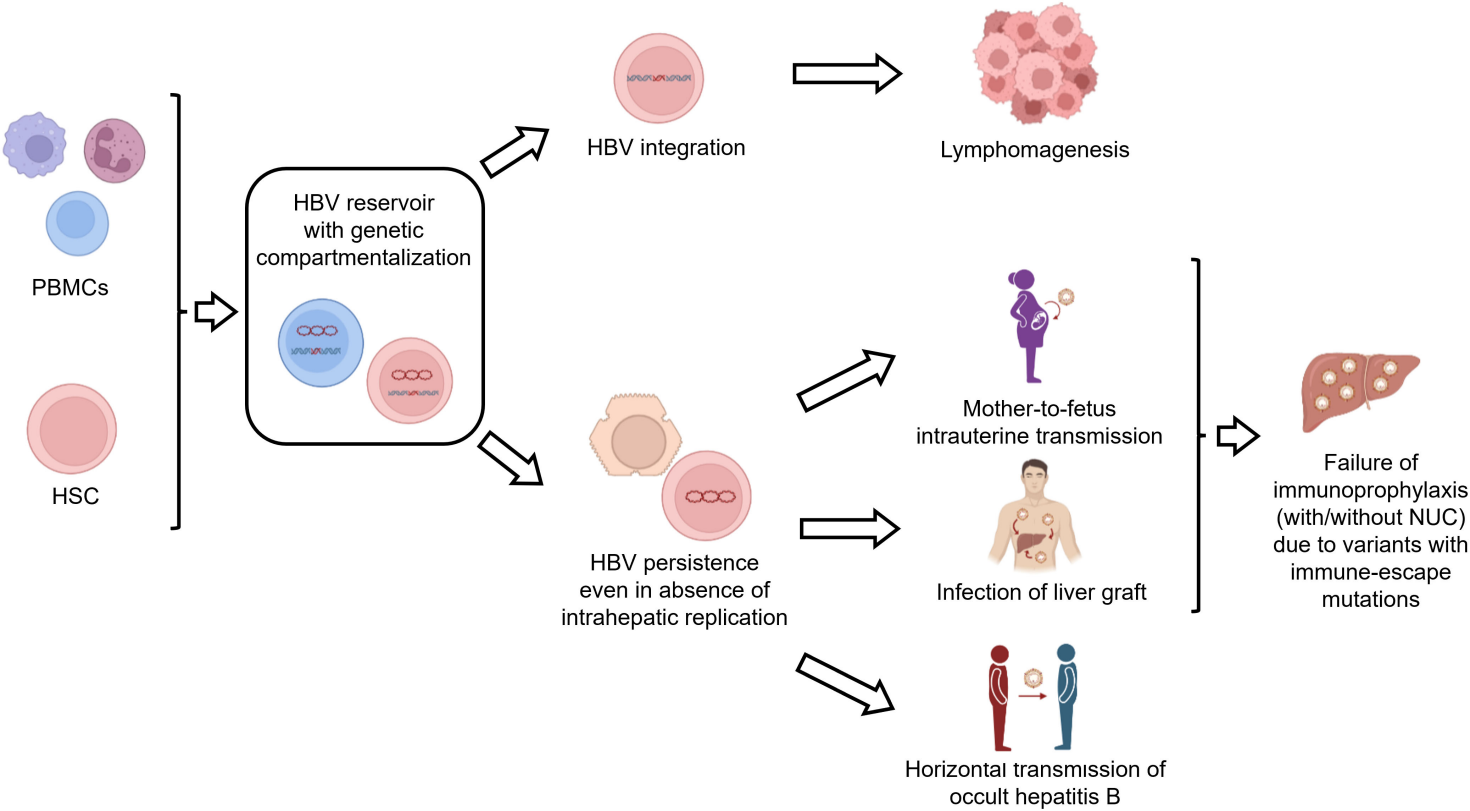
Consequences of HBV infection in lymphoid cells. The figure summarizes the implications of HBV infection in lymphoid cells. HBV-infected lymphoid cells can act as a reservoir of HBV infection. In particular, HBV persistence in lymphoid cells can contribute to i) the infection of the liver graft after liver transplantation, ii) intrauterine HBV transmission and iii) horizontal transmission of occult HBV infection. Furthermore, integrated HBV DNA can promote lymphomagenesis. Finally, the compartmentalization of viral variants with immune-escape mutations in PBMCs can contribute to the failure of vaccination or immunoglobulin treatment.

There is evidence that HBV infection in PBMCs can contribute to viral persistence even in the absence of viral replication in the liver (**Figure 3**). In this regard, a previous study in patients undergoing liver transplantation detected HBV DNA in the PBMCs of 7 out of 11 patients with no evidence of HBV replicative markers in the liver graft (74). Moreover, further studies have shown that PBMC-specific variants (not detected in the liver or serum before transplantation) can become the predominant viral variant after liver transplantation, thus contributing to the infection of the liver graft (75–78). Notably, some of these PBMC-specific variants can be characterized by the presence of immune-escape mutations (known to promote HBV evasion from neutralizing antibodies), thus contributing to the failure of immune-based therapy, typically used to prevent HBV reactivation after liver transplantation (75–78) (**Figure 3**). Overall findings support the role of PBMCs as a critical reservoir capable of fueling the persistence and spread of HBV infection.

Furthermore, HBV-infected PBMCs can serve as a source of intrauterine infection (79–81) (**Figure 3**). In this regard, a previous study has shown that HBV DNA-positive maternal PBMCs can cross the placental barrier and enter the fetal circulation, thus increasing the risk of vertical HBV transmission (81). Notably, these results raise the possibility of using HBV DNA detection in PBMCs as a biomarker of HBV intrauterine infection (81). Again, immune-escape mutations, detected in maternal HBV-infected PBMCs, have been shown to contribute to vertical HBV transmission despite immunoprophylaxis at birth (80). These findings support that HBV infection in PBMCs can lead to a genetic compartmentalization of viral variants playing an important role in promoting vertical HBV transmission (**Figure 3**).

HBV-infected PBMCs can also contribute to the horizontal transmission of HBV infection (**Figure 3**). Indeed, using the woodchuck model of hepadnaviral infection, previous studies have found that HBV-infected PBMCs can act as an important reservoir of transmission-competent occult hepadnaviral infection that invariably infects the lymphatic system through parenteral or vertical routes but rarely infects the liver (82, 83). This is in keeping with further studies conducted in humans showing the horizontal transmission (between family members) of occult HBV infection, characterized by a unique genotype and immune-escape mutations within the PBMC compartment (84, 85), again corroborating the role of genetic compartmentalization in PBMCs in mechanisms modulating HBV persistence and spreading.

In a similar direction, unique HBV strains, detected only in PBMCs, have been detected in HIV-infected patients, leading to HBV reactivation and fulminant hepatic failure (75, 86).

Overall findings support the role of PBMCs as a reservoir of HBV infection, critical to fueling human-to-human transmission by different routes and promoting HBV persistence. HBV genetic compartmentalization in PBMCs can further enhance HBV pathobiological properties. The role of HBV-infected PBMCs as a predictive biomarker of liver reinfection after transplantation and of increased risk of vertical transmission deserves further investigation for optimized management of patients with HBV infection.

## Potential implications of HBV lymphotropism for antiviral treatment and for HBV cure

The ideal treatment goal of anti-HBV treatment is the achievement of an HBV functional cure, defined as sustained HBsAg loss along with undetectable serum HBV DNA off-therapy, reflecting the silencing of cccDNA transcriptional activity (87–89). Indeed, HBsAg loss has been associated with a significant decrease in the risk of developing end-stage liver diseases, such as HCC and cirrhosis (90–92). Unfortunately, this goal is rarely achieved by currently available treatment options, including nucleos(t)ide analogs (NUCs) and peg-interferon alpha, with very limited or no impact on the cccDNA pool and its transcriptional activity.

Recently, experimental and clinical research in the field of HBV treatment has been devoted to the identification of novel drug molecules capable of promoting HBV functional cure by dampening viral antigen production and enhancing the strength of anti-HBV immune responses (88). In particular, promising data for the achievement of HBV functional cure derive from novel immunotherapies based on checkpoint inhibitors (i.e. PD-1 inhibitors) and therapeutic vaccination, aimed to restore T-cell and B-cell mediated anti-HBV immune response of chronically infected patients and from the RIG-I or Toll-like receptor (TLR) agonists (such as TLR-7 and TLR-8 agonists), aimed to potentiate the activation of innate immunity to counteract HBV infection (93, 94). In this light, the achievement of an HBV functional cure cannot disregard the role of PBMCs as an extrahepatic reservoir of HBV infection that could contribute to persistent HBV production and jeopardize the success of novel therapeutic approaches.

Notably, accumulating evidence demonstrates that liver macrophages play a double-faced role in HBV infection and pathogenesis. Indeed, on one hand M1 macrophages are known to favor HBV clearance by direct antiviral effect and by producing cytokines with trans-activating effect on adaptive immunity, on the other hand M2 macrophages and their phenotype modulation by HBV has been recently demonstrated to favor viral persistence and its related immunopathogenesis (95–97). Furthermore, according to literature, M1 macrophages suppresses early HCC tumorigenesis by eliminating cancer cells while M2 macrophages promote cancer cells proliferation and invasion by suppressing the adaptive immune system. In this light, this differential role should be taken into account for the design of novel immunotherapies, based on macrophages, aimed at achieving HBV functional cure as well as at hindering HBV-related tumorigenesis, including not only hepatocarcinogenesis but also lymphomagenesis.

Moreover, it has been recently shown that HBV DNA integration can represent an important source of HBsAg even when the transcriptional activity of cccDNA has been completely silenced. In this regard, the occurrence of HBV DNA integration in PBMCs suggests that this HBV reservoir can participate along with the liver in the continuous production and release of HBsAg in the patient’s serum. This can contribute to the elevated burden of viral antigens and the subsequent exhaustion of the anti-HBV immune response typically observed in chronic HBV infection (thus far recognized as a barrier to HBV cure) (98, 99). Furthermore, HBsAg production from PBMC-integrated HBV DNA can challenge the role of HBsAg loss as a surrogate marker of HBV functional cure.

Overall findings support the importance of deepening the role of lymphoid cells as HBV reservoir and finely characterizing the intermediates of HBV replication in this compartment. In particular, the evaluation of the efficacy of these novel drugs should include the monitoring of HBV replication in lymphoid cells to verify whether the drug can induce cccDNA silencing in all compartments where the virus hides and replicates.

## Conclusions

Numerous studies have confirmed the possibility of finding HBV DNA inside cellular compartments outside the liver, such as the pancreas and kidney, with several data on cells of the hematopoietic system, such as circulating PBMCs, NK cells and bone marrow precursor stem cells. Little is known about the mechanisms of HBV entry into hematological cells; nevertheless, the role of PBMCs as an extrahepatic reservoir of HBV infection deserves further investigation, since it may contribute to persistent HBV production, potentially jeopardizing the success of the current and novel therapeutic approaches, aimed at achieving HBV functional cure.

Furthermore, it is possible that the presence of HBV in immune cell precursors can promote uncontrolled cell proliferation and, possibly, malignancy.

Data correlating HBV with other hematologic malignancies have been reported; however, they are not always confirmed and are often supported by limited studies. In particular, among hematological disorders so far investigated, a stronger association has been reported for B-cell NHL.

There are no clear pathogenetic studies investigating the connections between chronic HBV infection and the onset of lymphatic and/or hematological malignancy, and it is possible that HBV acts more as an inflammatory driver than as a direct carcinogen.

Nevertheless, it is known that HBV can frequently integrate its DNA into the genome of the infected cells. HBV DNA integration is recognized as a mechanism promoting neoplastic transformation by several mechanisms, including the direct dysregulation of oncogenes and onco-suppressors, the production of chimeric viral-human RNAs and proteins with transactivating properties, and the promotion of overall genome instability. Notably, HBV DNA integration represents a common phenomenon in NHL tissues, molecular data show its potential impact in carcinogenesis, and epidemiological evidence seems to confirm a possible correlation.

In conclusion, although many epidemiological and laboratory studies have been produced in recent years, this remains a field of study due to the possible benefits of therapeutic or prophylactic strategies involving antivirals and vaccines.

## Author contributions

LS and VS: study conception and design. VS, VM, RS, MI, LP, and SD: literature revision. LS, VS, VM, RS, MI, LP, and SD: draft manuscript preparation. LS and VS: final manuscript revision. All authors contributed to the article and approved the submitted version.
